# DV-Hop Algorithm Based on Multi-Objective Salp Swarm Algorithm Optimization

**DOI:** 10.3390/s23073698

**Published:** 2023-04-03

**Authors:** Weimin Liu, Jinhang Li, Aiyun Zheng, Zhi Zheng, Xinyu Jiang, Shaoning Zhang

**Affiliations:** 1 College of Mechanical Engineering, North China University of Science and Technology, Tangshan 063210, China; 2 HUIDA Sanitary Ware Co., Ltd., Tangshan 063000, China

**Keywords:** wireless sensor network, node localization, DV-Hop, multi-objective salp swarm algorithm

## Abstract

The localization of sensor nodes is an important problem in wireless sensor networks. The DV-Hop algorithm is a typical range-free algorithm, but the localization accuracy is not high. To further improve the localization accuracy, this paper designs a DV-Hop algorithm based on multi-objective salp swarm optimization. Firstly, hop counts in the DV-Hop algorithm are subdivided, and the average hop distance is corrected based on the minimum mean-square error criterion and weighting. Secondly, the traditional single-objective optimization model is transformed into a multi-objective optimization model. Then, in the third stage of DV-Hop, the improved multi-objective salp swarm algorithm is used to estimate the node coordinates. Finally, the proposed algorithm is compared with three improved DV-Hop algorithms in two topologies. Compared with DV-Hop, The localization errors of the proposed algorithm are reduced by 50.79% and 56.79% in the two topology environments with different communication radii. The localization errors of different node numbers are decreased by 38.27% and 56.79%. The maximum reductions in localization errors are 38.44% and 56.79% for different anchor node numbers. Based on different regions, the maximum reductions in localization errors are 56.75% and 56.79%. The simulation results show that the accuracy of the proposed algorithm is better than that of DV-Hop, GWO-DV-Hop, SSA-DV-Hop, and ISSA-DV-Hop algorithms.

## 1. Introduction

A wireless sensor network (WSN) is a wireless multi-hop communication network system composed of low-cost, low-power, and self-reconfigurable sensor nodes [1]. A WSN is a sensing network based on the self-organization structure. It is formed in a certain monitoring area with multiple sensor nodes through wireless communication technology, which has less computing, storage, and transmission capacity. Wireless sensor network technology has many advantages, such as low cost, scalability, reliability, and flexibility [2]. It is widely used in smart homes, target tracking, military security, underwater detection, and many other technical fields [3,4]. In these scenarios, the data collected and transmitted by sensors are often meaningless if they do not contain location information. Therefore, the problem of wireless sensor node location has become one of the important research topics in wireless sensor networks [5]. The detection of location becomes difficult due to the fluctuation of signals and noise in the environment. Many difficulties have been faced in location analysis [6].

In wireless sensor networks, sensor nodes are usually deployed randomly. GPS is the most accurate and perfect localization technology to solve this problem. The exact position coordinates of all nodes can be obtained directly. However, there are significant limitations to equipping each sensor node with GPS [7]. Firstly, the cost and power consumption of installing GPS modules on all sensors increases dramatically in large networks. Then, it is susceptible to interference in an environment with many obstacles, and the localization accuracy is not satisfactory. Finally, the energy consumption of sensor nodes is also a main challenge with more GPS modules [8,9]. One solution is to use several sensor nodes equipped with localization modules, combined with known information in the network, to calculate the location of unknown nodes, which is node localization technology [10,11,12,13]. Nodes equipped with localization modules are called beacon nodes. Other nodes whose location information is unknown are called unknown nodes.

Node localization can be divided into range-based and range-free according to different methods [14]. The range-based algorithm requires distance or angle information of nodes. Its localization accuracy is high, but it has high requirements for nodes and is susceptible to environmental interference [15,16]. This method requires additional ranging equipment, which inevitably increases the overall cost. Range-based algorithms are mainly based on angle of arrival (AOA), time of arrival (TOA), time difference of arrival (TDOA), and receive signal strength (RSSI) [17,18,19,20]. The range-free algorithm requires connectivity information between the unknown node and the beacon node. It has the features of low cost, low energy consumption, and simple implementation [21]. The localization accuracy of range-free algorithms is usually lower than that of range-based algorithms due to the lack of ranging [22]. There are mainly centroid algorithms, Approximate PIT (APIT), DV-Hop [23,24,25], etc.

The multi-objective salp swarm algorithm (MSSA) is a heuristic algorithm, and its model can search for both fixed and moving food sources. The MSSA can well approximate the Pareto frontier with high coverage and convergence. The DV-Hop localization algorithm was proposed by Dragos et al. [26]. It is a non-range distributed location algorithm, because its simple principle is widely used. However, the DV-Hop localization accuracy and stability are poor. Cui et al. believe that the DV-Hop algorithm cannot meet the requirements of high sensor localization accuracy in some scenarios [27]. Messous et al. believe that the accuracy of existing solutions is still unsatisfactory [28].

To solve this problem, this paper designs a DV-Hop algorithm based on an improved multi-objective salp swarm algorithm. Firstly, the four communication radii are used to refine the hop count. Secondly, the average hop distance introduces a weighting factor on the basis of the minimum mean-square error to reduce the error. Finally, the improved multi-objective salp swarm algorithm is used to optimize the third stage of DV-Hop. The rest of the paper is structured as follows. In Section 2, we introduce the DV-Hop algorithm and describe the principles of the single-objective and multi-objective salp swarm algorithm algorithms. Our proposed improved localization scheme is given in Section 3. Section 4 is a performance analysis comparing the proposed algorithm with the three algorithms. Finally, the paper is summarized in Section 5.

### Related Work

The DV-Hop algorithm has been improved by some scholars. It mainly locates nodes based on network connectivity and topology. Node localization consists of two steps: one is distance estimation, and the other is coordinate estimation. Some scholars adopt the weighting strategy in the distance estimation stage. For example, Zhang et al. proposed that the unknown node would normalize the hop distance of all the beacon nodes received so as to obtain its own hop distance [29]. Hou et al. introduced differential knowledge in the hop distance calculation, and the average hop distance of each node was calculated based on its own difference error [30]. Wang et al. used the inverse distance weighting method in the calculation of the average hop distance, and the beacon node that is far away from the unknown node was assigned a small weight, thus reducing the error of the average hop distance [31]. Chen et al. used the minimum mean-square error criterion to calculate the distance error between beacon nodes, and at the same time used the minimum mean-square error criterion to calculate the average hop distance of unknown nodes to form a double-weighted average hop distance [32]. Gui et al. believed that the estimated distance of the original DV-Hop is one of the important reasons affecting the error, so the checkout step was introduced in the DV-Hop algorithm to improve the localization accuracy. Based on this, a three-beacon node estimation distance algorithm was proposed to further improve the localization accuracy [33].

However, some scholars use intelligent algorithms to optimize DV-Hop, such as Bo et al., who applied GA to solve the localization problem of wireless sensor networks and proposed a population constraint strategy based on three beacon nodes to solve the feasible domain of the population [34]. Singh et al. used the 2D hyperbolic method to determine the unknown node location, and after that, PSO was used to correct the node location [35]. Kaur et al. replaced the original computation with the GWO algorithm in the calculation of average hop distance so that all beacon nodes could obtain the exact average hop distance [36]. Chai et al. designed a parallel WOA algorithm and introduced the tribal annexation communication strategy and the group psychological communication strategy in the parallel algorithm to enhance the population diversity of WOA and avoid local optimal solutions [37]. Li et al. proposed three parallel cat colony algorithms and applied them to solve the localization problem of wireless sensor networks, which greatly reduced the running memory and computationally optimized variables [38]. Sabahat et al. used the average position of beetles in the BAS algorithm and also introduced inertia coefficients to update the position. The application of the improved BAS to the localization problem of wireless sensor networks greatly improved the accuracy and stability of localization [39]. With the application of intelligent algorithms in wireless sensor network localization, some researchers use multi-objective optimization to solve the localization problem. For example, Wang et al. proposed a multi-objective DV-Hop algorithm based on NSGA-II, which changed the population constraint strategy based on three beacon nodes to a population constraint strategy based on all beacon nodes. The localization accuracy was improved [40]. Kanwar et al. combined six single-objective functions with three multi-objective functions and considered the effect of noise on the communication radius. The solution was performed using the multi-objective PSO algorithm and obtained good localization accuracy [41]. Huang et al. proposed to combine Manhattan and Euclidean to obtain new frequency hopping and hop distance, and used the NSGA-II algorithm for iterative optimization to improve localization accuracy and localization adaptability [42].

## 2. Methods

### 2.1. DV-Hop

In this section, we specifically introduce the implementation process of the DV-Hop algorithm. The traditional DV-Hop consists of three stages.

Phase 1: Connectivity detection and calculation of hop counts between each unknown node and beacon node.

Connectivity detection is performed to ensure that the nodes can be communicated. In the first stage, the initialization value of node hop count information is 0. Each beacon node broadcasts packets into the network with a radius *R* around itself. The hop counts increase by 1 for each packet forwarded. The node stores the minimum hops between itself and the beacon node.

Phase 2: Estimating the distance between the unknown node and the beacon node.

The minimum hop count obtained in the first stage is estimated using Equation (1) to estimate the average hop distance *Hopsize_i_*.

(1) $$Hopsize_{i}=\sum_{j\neq i}^{N}\sqrt{{\left(x_{i}-x_{j}\right)}^{2}-{\left(y_{i}-y_{j}\right)}^{2}}/\sum_{j\neq i}^{N}h_{ij}$$

where (*x_i_*, *y_i_*) and (*x_j_*, *y_j_*) are the coordinates of beacon nodes *i* and *j*, *h_ij_* is the minimum hop count between *i* and *j* (*i* ≠ *j*), and *Hopsize_i_* is the average hop distance from beacon node *i* to beacon node *j*. The unknown node takes the *Hopsize* received first as its average hop distance, and estimates the distance *d_i_* with each beacon node based on it. The calculation formula is shown in Equation (2).

*d_i_ = Hopsize_i_ × h_i_* (2)

where *h_i_* is the minimum hop count from unknown node to beacon *i*.

Phase 3: Calculation of unknown nodes coordinates.

Since the distance between an unknown node and each beacon node is estimated from Equation (2), the relationship between the beacon node and the unknown node is shown in Equation (3).

(3) $$\left\{ \begin{array}{c} {\left(x_{1}-x\right)}^{2}+{\left(y_{1}-y\right)}^{2}=d_{1}{}^{2}\\ {\left(x_{2}-x\right)}^{2}+{\left(y_{2}-y\right)}^{2}=d_{2}{}^{2}\\ \vdots \\ {\left(x_{n}-x\right)}^{2}+{\left(y_{n}-y\right)}^{2}=d_{n}{}^{2} \end{array}\right.$$

where (*x*, *y*) are the coordinates of the unknown nodes. Equation (3) can be transformed into Equation (4) by matrix.

(4) $$\left\{ \begin{array}{c} x_{1}{}^{2}-x_{n}{}^{2}+y_{1}{}^{2}-y_{n}{}^{2}-2x(x_{1}-x_{n})-2y(y_{1}-y_{n})=d_{1}{}^{2}-d_{n}{}^{2}\\ x_{2}{}^{2}-x_{n}{}^{2}+y_{2}{}^{2}-y_{n}{}^{2}-2x(x_{2}-x_{n})-2y(y_{2}-y_{n})=d_{2}{}^{2}-d_{n}{}^{2}\\ \vdots \\ x_{n-1}{}^{2}-x_{n}{}^{2}+y_{n-1}{}^{2}-y_{n}{}^{2}-2x(x_{n-1}-x_{n})-2y(y_{n-1}-y_{n})=d_{n-1}{}^{2}-d_{n}{}^{2} \end{array}\right.$$

Equation (4) can be written as *AX* = *B*, where *A*, *X*, and *B* are shown in Equations (5)–(7).

(5) $$A=2\left[\begin{array}{cc} \left(x_{1}-x_{n}\right) & \left(y_{1}-y_{n}\right)\\ \begin{array}{c} \begin{array}{c} \left(x_{2}-x_{n}\right)\\ \ldots \end{array}\\ \left(x_{n-1}-x_{n}\right) \end{array} & \begin{array}{c} \left(y_{2}-y_{n}\right)\\ \ldots \\ \left(y_{n-1}-y_{n}\right) \end{array} \end{array}\right]$$

(6) $$X=\left[\begin{array}{c} x\\ y \end{array}\right]$$

(7) $$B=\left[\begin{array}{c} x_{1}{}^{2}-x_{n}{}^{2}+y_{1}{}^{2}-y_{n}{}^{2}+d_{n}{}^{2}-d_{1}{}^{2}\\ \begin{array}{c} x_{2}{}^{2}-x_{n}{}^{2}+y_{2}{}^{2}-y_{n}{}^{2}+d_{n}{}^{2}-d_{2}{}^{2}\\ \vdots \\ x_{n-1}{}^{2}-x_{n}{}^{2}+y_{n-1}{}^{2}-y_{n}{}^{2}+d_{n}{}^{2}-d_{n-1}{}^{2} \end{array} \end{array}\right]$$

Let *F*(*X*) = ||*AX*-*B*||^2^ and let *F*′(*X*) = 0. As shown in Equations (8) and (9),

(8) $$\frac{\partial f(x)}{\partial x}=\frac{\partial }{\partial x}∥AX-B{∥}^{2}=2A^{T}(AX-B)=2(A^{T}AX-A^{T}B)$$

(9) $$A^{T}AX=A^{T}B$$

The location of unknown nodes is estimated by Equation (10):

*X* = (*A^T^A*)^−1^*A^T^B* (10)

### 2.2. Multi-Objective Salp Swarm Algorithm

#### 2.2.1. Single-Objective Salp Swarm Algorithm

In the salp swarm algorithm (SSA), *F*(*x*) is denoted as the objective function, and {*ul*} is the optimal solution found by the algorithm that matches the objective function.

(11) $$\mathrm{Minisize}/\mathrm{Maxisize}:F\left(x\right)=\left\{f_{1}\left(x\right)\right\}$$

(12) $${\mathrm{Subject}\;\mathrm{to}:\;g}_{i}(x)\geq 0,h_{k}(x)=0$$

(13) $$lb_{j}\leq x_{j}\leq ub_{j}$$

where *i* and *k* are the number of constraints on the inequality and equation, respectively; *lb_j_* represents the lower bound on the *j*th variable; and *ub_j_* represents the upper bound on the *j*th variable.

In SSA, the salp chain is composed of leaders and followers. The leader is at the front of the salp chain, while other individuals are followers [43]. The random initialization population formula is shown in Equation (14):

*X_N_*_×*d*_ = rand (*N*, *d*) × (*ub* − *lb*) + *lb* (14)

where *N* is the population number and *d* is the dimension, *ub* is the upper bound, *lb* is the lower bound, and rand (*N*, *d*) is a random array of *N* rows and *d* columns between [0, 1].

In SSA, the location of the food source is the target location of all salps. It is the global optimal solution in the exploration process and affects the leader position update. The leader position update formula is as follows:

(15) $$x_{j}^{i}=\left\{ \begin{array}{l} F_{j}+c_{1}((ub_{j}-lb_{j})c_{2}+lb_{j}),c_{3}\geq 0.5\\ F_{j}-c_{1}((ub_{j}-lb_{j})c_{2}+lb_{j}),c_{3}\leq 0.5 \end{array}\right.$$

where $x_{j}^{i}$ is the position of the *i*th leader in the *j*th dimension, *F_j_* is the location of the food source in the *j*th dimension; *ub_j_* is the upper bound in the *j*th dimension; *lb_j_* is the lower limit in the *j*th dimension; and *c*_1_, *c*_2_, with *c*_3_ are random numbers.

In terms of Equation (15), it can be seen that the leader position update is mainly influenced by the food source position. Parameter *c*_1_ is defined as follows.

(16) $$c_{1}=2\times \exp(-{\left(4t/T_{max}\right)}^{m})$$

where *t* is the current number of iterations, the power factor *m* = 2, and *Tmax* is the maximum number of iterations. The parameter *c*_1_ decreases adaptively during the iterations. It contributes to the exploration ability when the value is relatively large, and it helps with specific development capabilities when the value is small. *c*_1_ can make the exploration and exploitation ability of the SSA in a good state. Thus, *c*_1_ is the most important parameter in the SSA.

To update the position of followers, the following formula is used.

(17) $$x_{j}^{i}=at^{2}/2+v_{0}\Delta t$$

where *i* ≥ 2, *x^i^_j_* is the position of the *i*th follower in the *j*th dimension, Δ*t* is time, *v*_0_ is the initial velocity, *a* = (*v_t_* − *v*_0_)/Δ*t*, *v_t_* = (*x^i^_j_* − *x_j_^i^*^−1^)/Δ*t*, and *x_j_^i^*^−1^ is the position of the (*i* − 1)st salp in the *j*th dimension. Since time is the difference between the number of iterations, Δ*t* = 1, and the initial velocity *v*_0_ = 0. Equation (18) can be expressed as:

(18) $$x_{j}^{i}=(x_{j}^{i}+x_{j}^{i-1})/2$$

With Equations (15) and (18), the salp chains can be simulated.

#### 2.2.2. Multi-Objective Salp Swarm Algorithm

A multi-objective optimization problem deals with multiple objectives simultaneously, and all objectives are to be optimized. It can be expressed as:

(19) $$\mathrm{Minisize}/\mathrm{Maxisize}:F\left(x\right)=\left\{f_{1}\left(x\right),f_{2}\left(x\right),\;\ldots ,f_{n}\left(x\right)\right\}$$

(20) $${\mathrm{Subject}\;\mathrm{to}:\;g}_{i}(x)\geq 0,h_{k}(x)=0$$

(21) $$lb_{j}\leq x_{j}\leq ub_{j}$$

where *n* is the number of objectives; *i* and *k* are the number of constraints on the inequality and equation, respectively; *lb_j_* represents the lower bound on the *j*th variable; and *ub_j_* represents the upper bound on the *j*th variable.

The multi-objective problem cannot be solved by the SSA. The main reason is that the solution to the multi-objective problem is a group of solutions called the Pareto-optimal set. The SSA can drive salps close to the food source and update it in the iterative process. However, the multi-objective problem cannot be addressed by this algorithm. The main reason is that the SSA only saves one solution as the optimal solution.

In the MSSA, a food repository is equipped to solve the problem. This repository stores the best solutions obtained during the optimization process. The capacity of the repository storing optimal solutions is limited. Each salp is compared with all repository original solutions using the Pareto dominance operator in the optimization process. The comparison rules are as follows.

(1) If a salp is superior in the repository, then that salp should be put into the repository, and the original solution should be taken out. If a salp is superior to a group of solutions in the repository, then that group of solutions should be removed from the repository, and the salp should be added to the repository.
(2) If there is at least one original solution in the repository that is superior to that salp, then that salp should be discarded and not added to the repository.
(3) If the salp is not superior to all repository residents, the salp is the optimal solution and must be added to the repository.
(4) If the repository is full and salp is not superior to the repository’s original solution, a distance *d* for calculating the neighboring solution numbers is introduced at this time. As shown in Equation (22). The number of neighboring solutions is calculated, and the roulette wheel selection strategy is used to select the solution with a high number of neighboring solutions for deletion.
  (22) $$\vec{d}=(\vec{max}-\vec{min})/\begin{array}{cc} repository & size \end{array}$$
  where $\vec{min}$ and $\vec{max}$ are the minimum and maximum fitness values in the population, respectively; and *repository size* is the number of current repositories.

In the food selection stage, there is more than one optimal solution in the multi-objective search space. The appropriate approach is to select the least crowded region from a set of optimal solutions. This can be achieved using the same sorting procedure used in the repository maintenance operator and roulette wheel selection. The main difference is the probability of selecting the optimal solution. The higher the rank of the solution in the repository maintenance deletion, the more likely it is to be selected. In contrast, the lower the rank for the optimal solution in the repository, the more likely it is to be selected as a food source.

## 3. Our Proposed IMSSA-DV-Hop Scheme

### 3.1. Error Analysis

Node location is the problem of obtaining the absolute coordinates of nodes in wireless sensor networks. In the DV-Hop algorithm, the distance between nodes is obtained by multiplying hop counts by the average hop distance. During the hop count calculation, all nodes within the node communication radius are recorded as 1. However, the distances between nodes are different, which leads to a large error. The unknown node receives the average hop distance of the nearest beacon node to it and takes that average hop distance as its own, which leads to an increase in the localization error. These methods are inherently inaccurate and sensitive to bias when solving for unknown node coordinates by least squares or maximum likelihood estimation.

Based on the above analysis, we have made a series of improvements to the DV-Hop algorithm, and the improved multi-objective salp swarm DV-Hop algorithm (IMSSA-DV-Hop) is proposed.

### 3.2. Subdivision Hop Count

Nodes within the node communication radius are noted as 1, but the distance between nodes is not the same, which leads to a large error. Thus, the minimum hop count is subdivided again. As shown in Equation (23).

(23) $$Hopsize_{min}=\left\{ \begin{array}{l} 1/m,0<dis<R/m\\ 2/m,R/m<dis<2\times R/m\\ \vdots \\ k/m,(k-1)\times R/m<dis<k\times R/m,k=1,2,\cdots ,m \end{array}\right.$$

Obviously, the division of the hop count becomes more accurate as *m* becomes large, and the calculation error becomes smaller. However, the larger the value of *m* taken, the higher the requirement for sensor nodes, and the cost rises. With this in mind, *m* = 4 is used in this paper.

### 3.3. Beacon Node Average Hop Distance Correction

In the DV-Hop algorithm, the unknown node takes the average hop distance from the nearest beacon node as its own average hop distance to calculate the distance. However, the network structure is random, and the hop distance from the unknown node to each beacon node is not the same, so the error is large.

First, the average hop distance of the beacon nodes is improved. In the DV-Hop algorithm, the calculation of the average hop distance is based on the unbiased estimation criterion. That is, it is obtained by Equation (24).

(24) $$f_{i}=(1/N-1)\sum_{j\neq i}\left(d_{ij}-Hopsize_{i}\times hop_{i,j}\right)$$

where *N* is the number of beacon nodes, *Hopsize_i_* is the average hop distance of beacon node *i*, and *hop_i,j_* is the minimum number of hops between beacon nodes *i* and *j*, and *f_i_* is the cost function of the *i*th node.

The measurement error follows the Gaussian distribution. According to the parameter estimation theory, it is more reasonable to use the mean-square error than the variance only. Therefore, the mean-square error is used to calculate the average hop distance of the beacon node. Equation (25) can be obtained by transforming Equation (24):

(25) $$f_{i}={\sum_{j\neq i}\left(d_{ij}-Hopsize_{i}\times hop_{i,j}\right)}^{2}$$

According to the calculation rule of unbiased estimation, take the first-order derivative of Equation (25) and set it as 0 to obtain the average hop distance conforming to the minimum mean-square error, as shown in Equation (26):

(26) $$Hopsize_{i}=\sum_{j\neq i}hop_{i,j}\times d_{i,j}/\sum_{j\neq i}hop_{i,j}{}^{2}$$

Beacon nodes with different distances from the unknown nodes reflect the local network state differently. The close beacon nodes can reflect the actual average hop distance of the nodes more accurately. Therefore, a large weight is assigned to the close beacon nodes. It is required to consider the average hop distance of multiple beacon nodes to estimate the average hop distance more accurately. The weight value formula is shown in Equation (27).

(27) $$w_{i}=1/hop_{ij}/\sum_{i=1}^{N}1/hop_{ij}$$

where *hop_ij_* is the hop count from the unknown node to the beacon node, and *w_i_* is the weighted correction factor of the hop distance of the unknown node. The average hop distance of the unknown node can be solved according to Equation (28).

(28) $$Hopsize_{u}=\sum_{i=1}^{N}w_{i}\times Hopsize_{i}$$

### 3.4. Multi-Objective Model

In the DV-Hop algorithm, the initial objective function is shown in Equation (29).

(29) $$fitness_{1}=\min(\sum_{i=1}^{m}|\sqrt{{\left(x_{i}-x\right)}^{2}+{\left(y_{i}-y\right)}^{2}}-d_{i}|)$$

where *d_i_* is the estimated distance between beacon node *i* and the unknown node, (*x_i_*, *y_i_*) are the coordinates of beacon node *I*, and (*x*, *y*) are the coordinates of the unknown node.

However, because *d_i_* is a constant obtained in the second stage of the algorithm, the position calculated with *d_i_* is not the actual position, but it is close to the estimated position. Therefore, another objective function needs to be added to enhance the search constraint. The estimated distance *d_i_* in the original objective function is replaced with the theoretical distance. This results in a new objective function, as shown in Equation (30).

(30) $$fitness_{2}=\min(\sum_{i=1}^{m}|\sqrt{{\left(x_{i}-x\right)}^{2}+{\left(y_{i}-y\right)}^{2}}-d_{it}|)$$

where *d_it_* is the theoretical distance from the unknown node *t* to the beacon node *i*. As shown in Equation (31).

*d_it_ = div_as_ × h_it_* (31)

where *h_it_* is the minimum hop count between the unknown node *t* and the beacon node *i*, and *dis_av_* is the theoretical value of the average per hop distance from the unknown node to the beacon node. Variable *dis_av_* as shown in Equation (32).

(32) $$dis_{av}=\int_{0}^{R}2\pi r^{2}dr/\int_{0}^{R}2\pi rdr=2R/3$$

### 3.5. Improved Multi-Objective Salp Swarm Algorithm

#### 3.5.1. Initialization

In this paper, a good point-set initialization strategy is used to optimize the multi-objective salp swarm algorithm, which is based on the following principle [44]. *G_S_* is the unit cube in *s*-dimensional Euclidean space; if *r* ∈ *G_S_*, then:

(33) $$P_{n}(k)=\{(\{r_{1}{}^{\left(n\right)}\cdot k\},\{r_{2}{}^{\left(n\right)}\cdot k\},\cdots \{r_{3}{}^{\left(n\right)}\cdot k\}),1\leq k\leq n\}$$

Deviation *Φ* (*n*) satisfies *Φ* (*n*) = *C* (*r*, *ε*) *n*^−1+*ε*^, where *C* (*r*, *ε*) *n*^−1+*ε*^ is a constant related to *r* and *ε* only (*ε* is an arbitrary positive number). Then, *P_n_* (*k*) is said to be a good point-set, *r* is a good point, and {*r_s_*^(*n*)^**·***k*} represents the fractional part and *n* denotes the number of points, *r* = {2cos(2π*k*/*p*), 1 ≤ *k* ≤ *n*} (*p* is the smallest prime number satisfying(*p* − 3)/2 ≥ *s*). Map it to a search space as:

*x_i_* (*j*) = (*ub_j_* − *lb_j_*) · {*r_j_^i^* · *k*} + *lb_j_* (34)

where *ub_j_* and *lb_j_* denote the upper and lower bounds of the *j*th dimension.

#### 3.5.2. Fuzzy Selection

After obtaining the Pareto-optimal solution set, the best solution and the solution to be deleted are selected in the repository by a fuzzy selection mechanism. *u_i_* is denoted as the membership of the *i*th objective function of the solution. As shown in Equation (35).

(35) $$\mu_{i}=\left\{ \begin{array}{l} 1,F_{i}\leq F_{i}^{min}\\ \left(F_{i}^{max}-F_{i}\right)/\left(F_{i}^{max}-F_{i}{}^{min}\right),F_{i}{}^{min}\leq F_{i}\leq F_{i}^{max}\\ 0,F_{i}\geq F_{i}^{max} \end{array}\right.$$

(36) $$u^{k}=\sum_{i=1}^{N_{obj}}\mu_{i}^{k}/\sum_{k=1}^{M}\sum_{i=1}^{N_{obj}}\mu_{i}^{k}$$

where *M* is the number of non-dominated solutions, *Nob_j_* is the number of objective functions, and $\mu_{i}^{k}$ is denoted as the membership of the *i*th objective function of the *k*th solution. The solution is judged according to the size of *u^k^*.

#### 3.5.3. Leader Position Updates Strategy

(1) Parameter adjustment

In the MSSA, *c*_1_ affects the search capability of the algorithm, and its Equation (37) is as follows.

(37) $$c_{1}=2e^{-{\left(4l/L\right)}^{m}}$$

In the original algorithm, *m* = 2. However, we found that *c*_1_ in the [0.05, 0.95] provides good results in initial phase exploration and in final phase development. Therefore, the lower *c_min_* and upper *c_max_* of the control parameter *c*_1_ are in the [0.05, 0.95], and the adaptive equation is shown in Equation (38).

(38) $$c_{1}=c_{max}+(c_{min}-c_{max})\times \log_{10}(a+10t/t_{max})$$

where *c* is the inertia weight parameter and *a* is a random number between [0, 1], and *t* and *t_max_* are the current and maximum number of iterations.

(2) Adaptive weight

The weight factor is added for food, and the influence of food source on the leader gradually decreases with the increase of iterations. Local extremes are avoided in the early stages of convergence. Convergence late approximates the optimal value and achieves high solution accuracy. The weight factor of food addition is shown in Equation (39):

(39) $$w=(w_{max}-w_{min})\times {\left(l/L\right)}^{2}$$

where *w_max_* is the maximum inertia weight, *w_min_* is the minimum inertia weight, *l* is the number of current iterations, and *L* is the total number of iterations. *w_max_* = 0.9 and *w_min_* = 0.4 have the best performance. As the iterations proceed, the inertia weight decreases linearly from 0.9 to 0.4.

(3) Levy flight strategy

Lévy flight obeys the Lévy distribution, which is a movement between the short-distance search followed by an occasional longer-distance walk [45]. The position update equation for the Levy flight is shown in Equation (40).

(40) $$L(s)\sim {\left|s\right|}^{-1-\beta },\;\;\;\;\;\;\;\;0<\beta \leq 2$$

where *s* is the random step size. Since the Lévy flight is very complex, the algorithm proposed by Mantegna is used in this paper to calculate the random step size, as shown in Equation (41)

(41) $$s=\mu /{\left|\nu \right|}^{1/\beta }$$

In the equation, *μ* and *υ* obey normal distribution.

(42) $$\left\{ \begin{array}{l} \mu ∼N(0,\sigma_{\mu }{}^{2})\\ v∼N(0,\sigma_{v}{}^{2}) \end{array}\right.$$

(43) $$\left\{ \begin{array}{c} \sigma_{\mu }={\left\{\Gamma (1+\beta )\sin(\pi \beta /2)/\Gamma [\frac{\left(1+\beta \right)}{2}]\times \beta \times 2^{(\beta -1)/2}\right\}}^{\frac{1}{\beta }}\\ \sigma_{\nu }=1 \end{array}\right.$$

The parameter *β* is 0 < *β* < 2, and generally *β* = 1.5.

In summary, the leader’s position update formula is shown in Equation (44):

(44) $$x_{j}^{i}=\left\{ \begin{array}{l} w\times F_{j}+c_{1}((ub_{j}-lb_{j})c_{2}+lb_{j})\times s,\;\;\;\;\;\;\;c_{3}\geq 0.5\\ w\times F_{j}-c_{1}((ub_{j}-lb_{j})c_{2}+lb_{j})\times s,\;\;\;\;\;\;\;c_{3}<0.5 \end{array}\right.$$

(4) Follower location update strategy

This section introduces the mayfly mating process formula in the mayfly algorithm to improve the follower position update formula [46]. Based on this, a follower update strategy with an adaptive mayfly search mechanism is proposed. The fitness values of the two individuals are compared and the fitness value that meets the multi-objective requirements of this paper is selected. The update position is biased to the side with good fitness, so the follower position update formula is as follows.

(45) $$x_{j}^{i}=\left\{ \begin{array}{l} \eta x_{j}^{i}+(1-\eta )x_{j}^{i-1},f(x_{j}^{i})>f(x_{j}^{i-1})\\ \eta x_{j}^{i-1}+(1-\eta )x_{j}^{i},f(x_{j}^{i})\leq f(x_{j}^{i-1}) \end{array}\right.$$

where *η* is the dynamic adaptive factor, as shown in Equation (46).

(46) $$\eta =0.8-0.2\times 1/\left(1+e^{-t}\right)$$

*t* is the current number of iterations.

### 3.6. IMSSA-DV-Hop Algorithm Flow Chart

**Figure 1:**
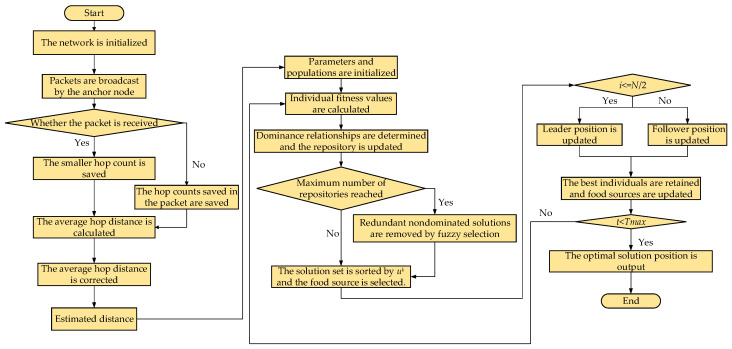
Flow chart of IMSSA-DV-Hop algorithm.

According to the proposed algorithm improvement strategy, the IMSSA-DV-Hop algorithm is proposed, and the flow chart is shown in Figure 1.

## 4. Experimental Results and Analysis

### 4.1. Experimental Environment and Evaluation Criteria

**Figure 2:**
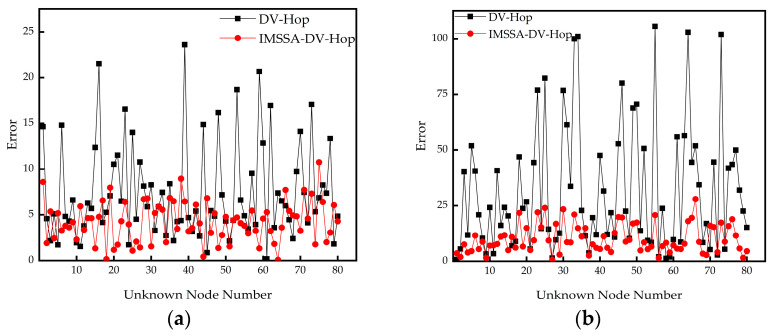
Ranging error diagrams: (**a**) square random topology; (**b**) C-shaped random topology.

To verify the effectiveness of the IMSSA-DV-Hop algorithm, this algorithm is tested and simulated on MATLAB 2016b on a computer configured with Intel (R) Core (TM) i7-7700HQ CPU @ 2.80 GHz processor (Intel, Santa Clara, CA, USA), 16 GB RAM and Windows 10 operating system. The proposed algorithm is firstly compared with the DV-Hop algorithm in square random topology and C-shaped random topology. The range error line diagram is shown in Figure 2.

As can be seen from Figure 2a,b, the IMSSA-DV-Hop algorithm is significantly improved compared with DV-Hop. Second, to verify the effectiveness of the proposed algorithm, a large number of simulation experiments are conducted with different communication radii, node numbers, beacon node numbers, and areas as constraints. The proposed algorithm is compared with the original DV-Hop algorithm, SSA-DV-Hop algorithm, GWO-DV-Hop algorithm, and ISSA-DV-Hop algorithm. To consider the cost of practical application, specific experimental parameters are shown in Table 1.

The normalized relative error equation is used as the index for comparison. The relative error equation after normalization is shown in Equation (47):

(47) $$error=\sum_{i=1}^{N}\sqrt{{\left(x_{0}-{\hat{x}}_{0}\right)}^{2}-{\left(y_{0}-{\hat{y}}_{0}\right)}^{2}}/(N\times R)$$

where $\left(x_{0},y_{0}\right)$ and $\left({\hat{x}}_{0},{\hat{y}}_{0}\right)$ are the real and estimated coordinates of the unknown node, and *N* indicates the number of unknown nodes.

**Figure 3:**
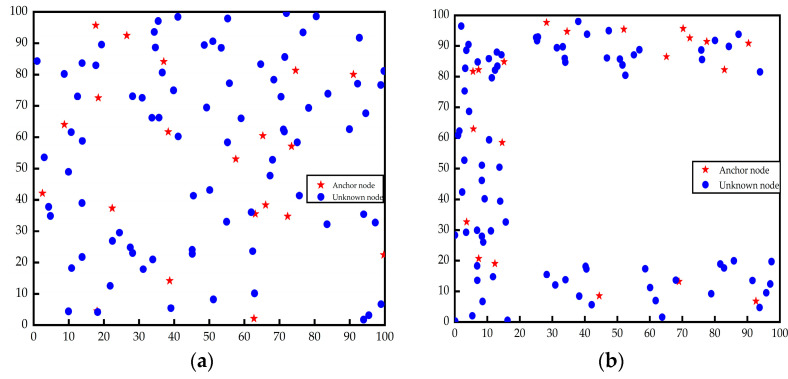
Node distribution diagrams: (**a**) square random topology; (**b**) C-shaped random topology.

The distribution of nodes in the square random topology and C-shaped random topology is shown in Figure 3a,b.

### 4.2. The Influence of Communication Radius on Localization Error

**Figure 4:**
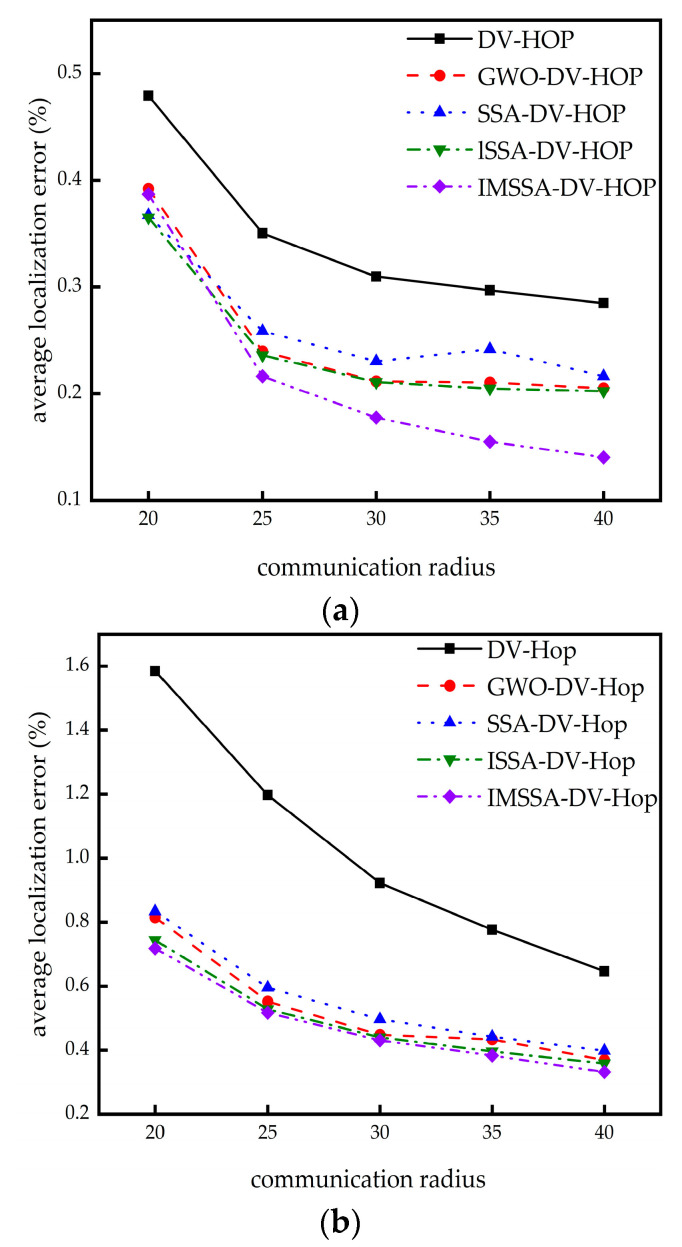
Localization error diagrams for different communication radius: (**a**) square random topology; (**b**) C-shaped random topology.

In this section, we research the influence of different communication radii on localization error. The node numbers and the beacon node numbers remain the same. At the same time, the communication radius is increased from 20 m to 40 m. The comparison results are shown in Figure 4.

As can be seen from Figure 4a, in the square random topology, the errors of the IMSSA-DV-Hop algorithm are close to those of the GWO-DV-Hop algorithm, when *R* is small and slightly higher than that of the SSA-DV-Hop algorithm and the ISSA-DV-Hop algorithm. However, with the increase in *R*, the localization errors decrease significantly. In the C-shaped random topology network, as shown in Figure 4b, the IMSSA-DV-Hop algorithm always has the minimum localization error regardless of the size of *R*.

The specific experimental data are shown in Table 2 and Table 3. The bolded data in Table are the optimal localization error values.

**Figure 5:**
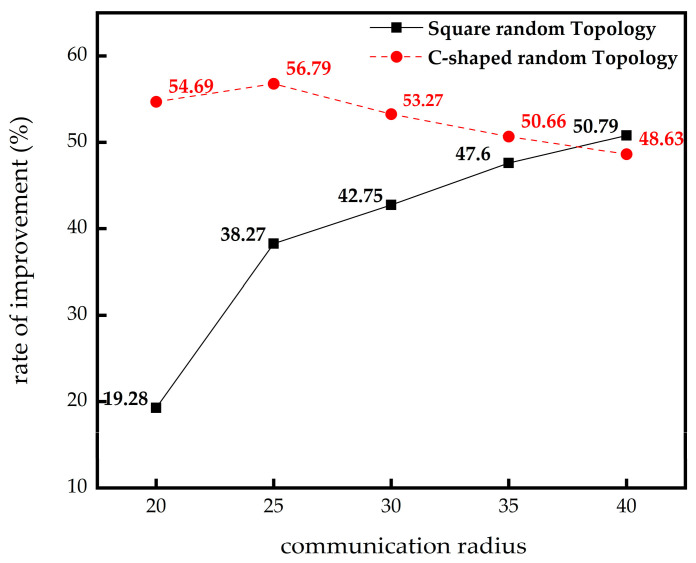
Comparison of localization error improvement in different communication radius.

It can be seen from Table 2 that in square random topology, compared with the DV-Hop algorithm, the IMSSA-DV-Hop algorithm reduces the localization errors by 19.28%, 38.27%, 42.75%, 47.60%, and 50.79%. It can be seen from Table 3 that in the C-shaped random topology, the errors are reduced by 54.69%, 56.79%, 53.27%, 50.66%, and 48.63%. The comparison of localization errors improvement is shown in Figure 5. Compared with the GWO-DV-Hop algorithm, the localization errors of the IMSSA-DV-Hop algorithm in the two topologies are increased by 31.64% and 11.88%.

### 4.3. The Influence of Node Numbers on Localization Error

**Figure 6:**
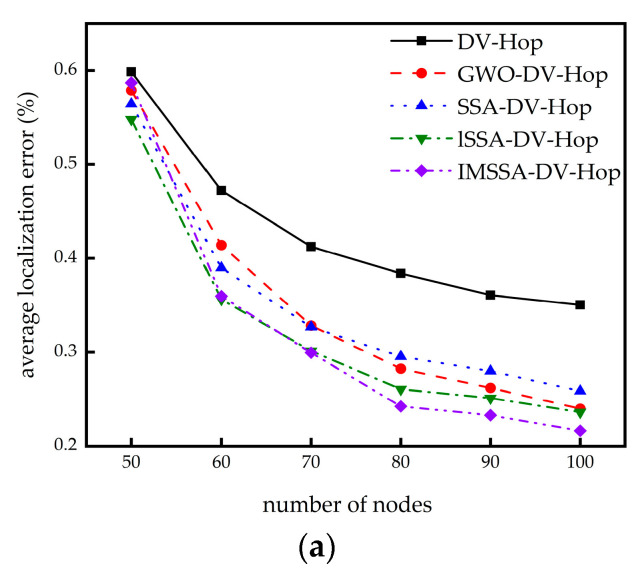

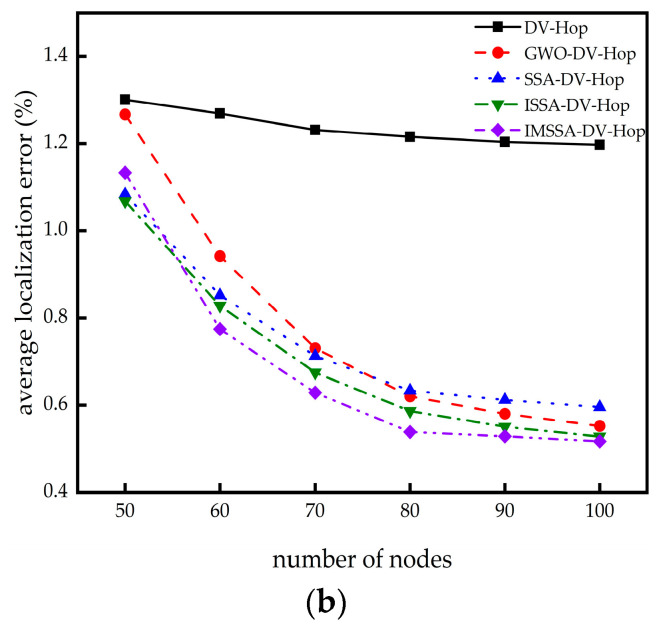
Localization error diagrams for different number of nodes: (**a**) square random topology; (**b**) C-shaped random topology.

In this section, we investigate the effect of different node numbers on the localization error. The communication radii and the beacon node numbers remain the same, while the node numbers are increased from 50 to 100. The comparison results are shown in Figure 6.

It can be seen from Figure 6a,b that in the two network topologies, the localization error of the IMSSA-DV-Hop algorithm is slightly greater than that of the three comparison algorithms when the node numbers are small. With the increase in the node numbers, the location errors of the IMSSA-DV-Hop algorithm improve significantly. No matter how the node numbers change, it is better than the DV-Hop algorithm.

The specific experimental data are shown in Table 4 and Table 5. The bolded data in Table are the optimal localization error values.

**Figure 7:**
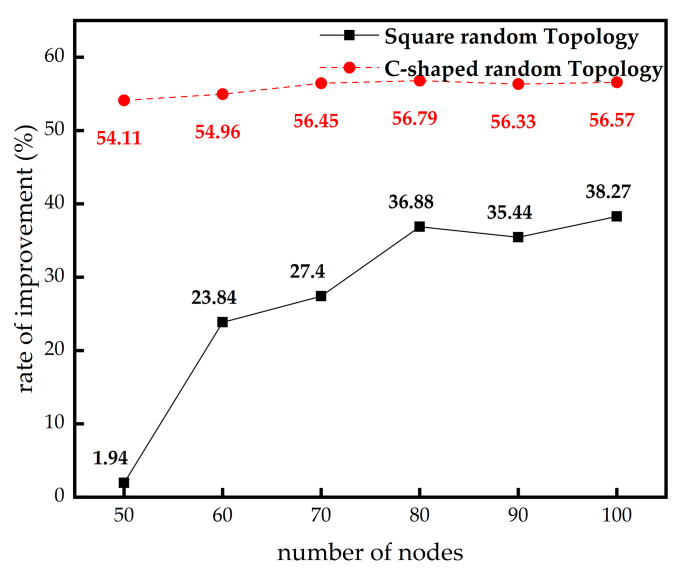
Comparison of localization error improvement in different number of nodes.

From Table 4, in the square random topology, compared with the DV-Hop algorithm, the IMSSA-DV-Hop algorithm reduces the localization errors by 1.94%, 23.84%, 27.40%, 36.88%, 35.44%, and 38.27%. From Table 5, In the C-shaped random topology, it reduces 54.11%, 54.96%, 56.45%, 56.79%, 56.33%, and 56.57%. The comparison of localization error improvement is shown in Figure 7.

### 4.4. The Influence of Beacon Node Numbers on Localization Error

**Figure 8:**
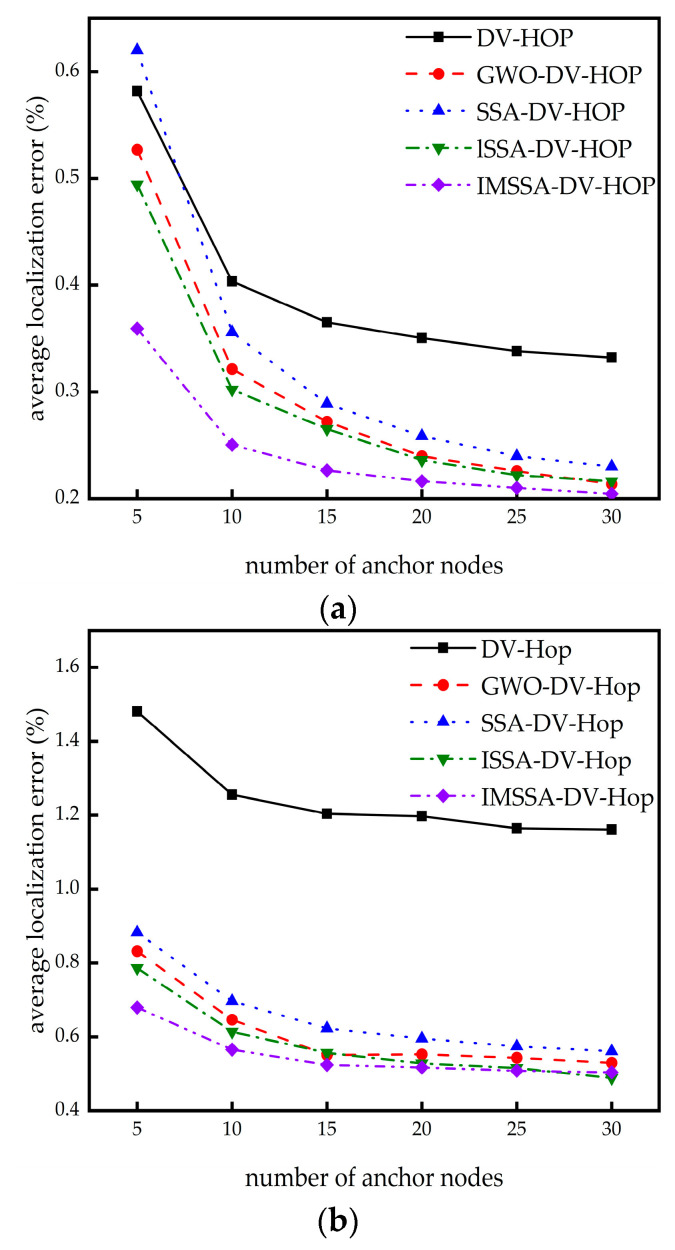
Localization error diagrams for different number of anchor nodes: (**a**) square random topology; (**b**) C-shaped random topology.

In this section, we research the influence of different beacon node numbers on localization error. The node numbers and communication radius remain the same, while the beacon node numbers are increased from 5 to 30. The comparison results are shown in Figure 8.

From Figure 8a,b, it can be seen that the IMSSA-DV-Hop algorithm in two random topologies can achieve lower localization error compared with the three algorithms compared in the case of fewer beacon nodes. In the C-shaped random topology, the performance of this algorithm is close to that of the ISSA-DV-Hop algorithm when the numbers of beacon nodes are large.

The specific experimental data are shown in Table 6 and Table 7. The bolded data in Table are the optimal localization error values.

**Figure 9:**
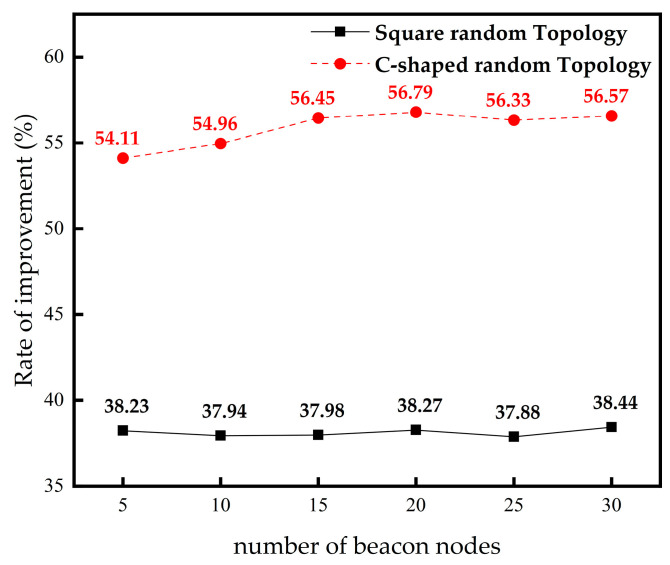
Comparison of localization error improvement in different number of anchor nodes.

From Table 6, in square random topology, compared with the DV-Hop algorithm, the localization errors of the IMSSA-DV-Hop algorithm are reduced by 38.23%, 37.94%, 37.98%, 38.27%, 37.88%, and 38.44%. From Table 7, In the C-shaped random topology, the localization errors are reduced by 54.11%, 54.96%, 56.45%, 56.79%, 56.33%, and 56.57%. The comparison of localization error improvement is shown in Figure 9. Compared with ISSA-DV-Hop, the localization errors of IMSSA-DV-Hop in the two topologies are increased by 27.31% and 13.59%.

### 4.5. The Influence of Area on Localization Error

**Figure 10:**
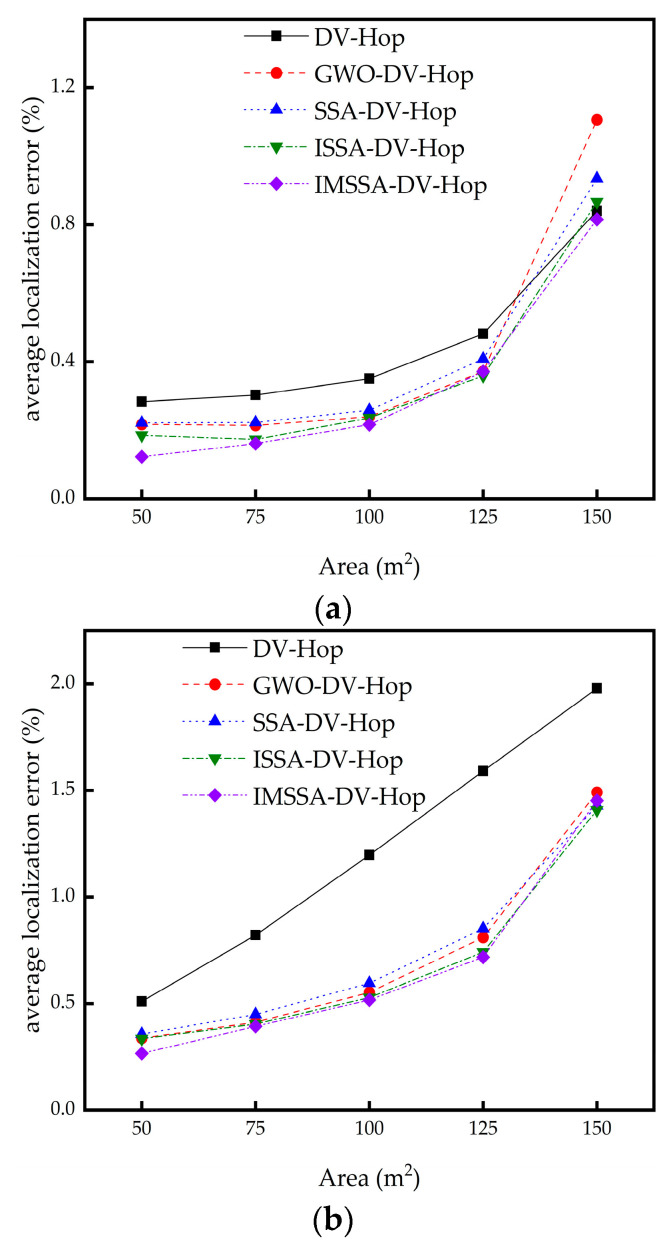
Localization error diagrams for different Area: (**a**) square random topology; (**b**) C-shaped random topology.

In this section, we study the influence of different regional areas on localization error. The area is increased from 50 × 50 m to 150 × 150 m. The comparison results are shown in Figure 10.

It can be seen from Figure 10a,b that the IMSSA-DV-Hop algorithm is significantly superior to the DV-Hop algorithm and three comparison algorithms in the two random topologies when the area is small at the initial stage. However, in the square topology structure, with the increase in the area, the localization errors of the proposed algorithm and the three comparison algorithms increase significantly. However, in the two topologies, the proposed algorithm is superior to the DV-Hop algorithm regardless of the size of the area. 

The specific experimental data are shown in Table 8 and Table 9. The bolded data in Table are the optimal localization error values.

**Figure 11:**
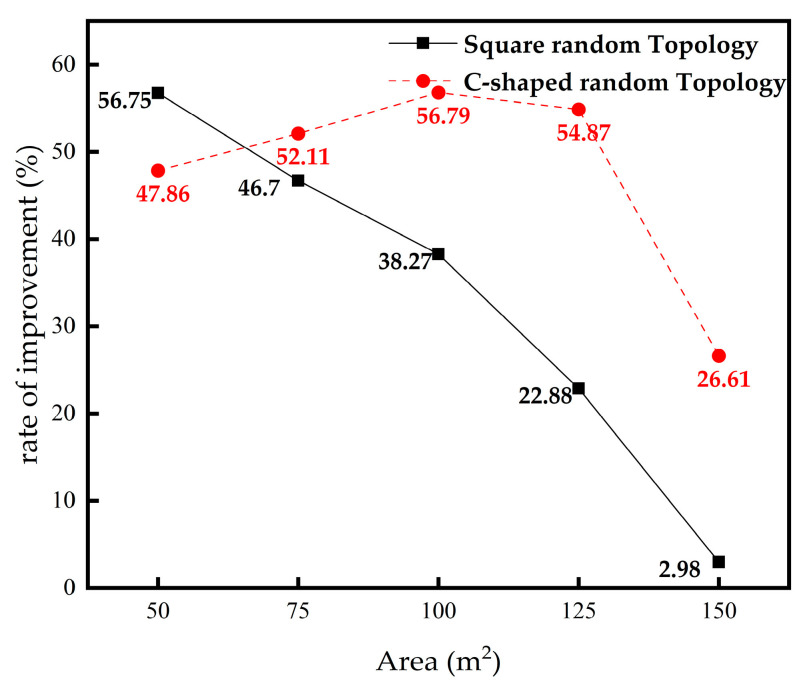
Comparison of localization error improvement in different Area.

As can be seen from Table 8, in the square random topology, the localization errors of the IMSSA-DV-Hop algorithm are reduced by 56.75%, 46.70%, 38.27%, 22.88%, and 2.98%, compared with the DV-Hop algorithm. As can be seen from Table 9, localization errors in C-shaped random topology are reduced by 47.86%, 52.11%, 56.79%, 54.87%, and 26.61%. The comparison of localization error improvement is shown in Figure 11.

## 5. Conclusions

In this paper, we propose an IMSSA-DV-Hop localization algorithm, which uses a multi-objective model based on the DV-Hop single-objective model to reduce the localization error. The first stage of traditional DV-Hop adopts subdivide hop count, average hop distance based, and minimum mean-square error weighting to reduce the errors in the first two stages of the DV-Hop algorithm and improve the localization accuracy. In IMSSA, the initial population of a good point-set is used to facilitate getting rid of local optimal solutions. Additionally, replacing the selection mechanism in the multi-objective salp swarm algorithm with fuzzy selection well selects the desired non-dominated solutions in the repository. In addition, the Levy flight strategy and the floating algorithm position update formula are used in the leader and follower position update, respectively, which improves the search efficiency of the algorithm and reduces the localization error. Experiments are conducted under two network topologies, and the experimental results show that the IMSSA-DV-Hop algorithm outperforms DV-Hop, GWO-DV-Hop, SSA-DV-Hop, and ISSA-DV-Hop.

## Figures and Tables

**Table 1:** Experimental parameter settings.

Parameter	Value
Communication radius (*R*)	25 m
Nodes	100
Beacon nodes	20
Area	100 × 100 m

**Table 2:** Localization error in different communication radius in square random topology.

Communication Radius		20	25	30	35	40
Square random topology	DV-Hop	0.4793	0.3504	0.3097	0.2958	0.2849
	GWO-DV-Hop	0.3921	0.2397	0.2116	0.2105	0.2051
	SSA-DV-Hop	0.3673	0.2587	0.2304	0.2417	0.2162
	ISSA-DV-Hop	**0.3651**	0.2360	0.2109	0.2048	0.2024
	IMSSA-DV-Hop	0.3869	**0.2163**	**0.1773**	**0.1550**	**0.1402**

**Table 3:** Localization error in different communication radius in C-shaped random topology.

Communication Radius		20	25	30	35	40
C-shaped random topology	DV-Hop	1.5847	1.1970	0.9225	0.7766	0.6467
	GWO-DV-Hop	0.8149	0.5525	0.4484	0.4340	0.3692
	SSA-DV-Hop	0.8335	0.5957	0.4966	0.4423	0.3981
	ISSA-DV-Hop	0.7438	0.5283	0.4402	0.3963	0.3589
	IMSSA-DV-Hop	**0.7181**	**0.5172**	**0.4311**	**0.3832**	**0.3322**

**Table 4:** Localization error in different number of nodes in square random topology.

Number of Nodes		50	60	70	80	90	100
Square random Topology	DV-Hop	0.5986	0.4723	0.4124	0.3839	0.3608	0.3504
	GWO-DV-Hop	0.5789	0.4141	0.3284	0.2824	0.2620	0.2397
	SSA-DV-Hop	0.5644	0.3897	0.3268	0.2954	0.2798	0.2587
	ISSA-DV-Hop	**0.5479**	**0.3562**	0.3014	0.2606	0.2511	0.2360
	IMSSA-DV-Hop	0.5870	0.3597	**0.2994**	**0.2423**	**0.2329**	**0.2163**

**Table 5:** Localization error in different number of nodes in C-shaped random topology.

Number of Nodes		50	60	70	80	90	100
C-shaped random Topology	DV-Hop	1.3009	1.2689	1.2315	1.2156	1.2036	1.1970
	GWO-DV-Hop	1.2668	0.9418	0.7309	0.6203	0.5806	0.5525
	SSA-DV-Hop	1.0839	0.8523	0.7131	0.6338	0.6122	0.5957
	ISSA-DV-Hop	**1.0680**	0.8284	0.6751	0.5868	0.5509	0.5283
	IMSSA-DV-Hop	1.1330	**0.7749**	**0.6289**	**0.5389**	**0.5288**	**0.5172**

**Table 6:** Localization error in different number of anchor nodes in square random topology.

Number of Beacon Nodes		5	10	15	20	25	30
Square random Topology	DV-Hop	0.5817	0.4035	0.3652	0.3504	0.3382	0.3322
	GWO-DV-Hop	0.5269	0.3213	0.2721	0.2397	0.2258	0.2136
	SSA-DV-Hop	0.6200	0.3559	0.2889	0.2587	0.2397	0.2301
	ISSA-DV-Hop	0.4943	0.3021	0.2652	0.2360	0.2220	0.2162
	IMSSA-DV-Hop	**0.3593**	**0.2504**	**0.2265**	**0.2163**	**0.2101**	**0.2045**

**Table 7:** Localization error in different number of anchor nodes in C-shaped random topology.

Number of Beacon Nodes		5	10	15	20	25	30
C-shaped random Topology	DV-Hop	1.4806	1.2556	1.2038	1.1970	1.1643	1.1610
	GWO-DV-Hop	0.8321	0.6464	0.5507	0.5525	0.5433	0.5298
	SSA-DV-Hop	0.8828	0.6969	0.6229	0.5957	0.5742	0.5611
	ISSA-DV-Hop	0.7864	0.6142	0.5568	0.5283	0.5160	**0.4888**
	IMSSA-DV-Hop	**0.6795**	**0.5655**	**0.5242**	**0.5172**	**0.5085**	0.5042

**Table 8:** Localization error in different Area in square random topology.

Area		50	75	100	125	150
square random Topology	DV-Hop	0.2833	0.3026	0.3504	0.4813	0.8404
	GWO-DV-Hop	0.2172	0.2139	0.2397	0.3717	1.2178
	SSA-DV-Hop	0.2214	0.2227	0.2587	0.4085	0.9347
	ISSA-DV-Hop	0.1854	0.1730	0.2360	**0.3589**	**0.9519**
	IMSSA-DV-Hop	**0.1225**	**0.1613**	**0.2163**	0.3947	1.1674

**Table 9:** Localization error in different Area in C-shaped random topology.

Area		50	75	100	125	150
C-shaped random Topology	DV-Hop	0.5102	0.8216	1.1970	1.5913	1.9785
	GWO-DV-Hop	0.3378	0.4127	0.5525	0.8108	1.4908
	SSA-DV-Hop	0.3569	0.4494	0.5957	0.8523	1.4290
	ISSA-DV-Hop	0.3353	0.4045	0.5283	0.7418	**1.4082**
	IMSSA-DV-Hop	**0.2660**	**0.3935**	**0.5172**	**0.7181**	1.4520

## Data Availability

All data are included in the work. No additional data present.
